# Refusal of Cancer-Directed Surgery Strongly Impairs Survival of Patients with Localized Hepatocellular Carcinoma

**DOI:** 10.1155/2010/381795

**Published:** 2010-12-20

**Authors:** Jue Wang, Fen Wei Wang

**Affiliations:** ^1^Department of Internal Medicine, Section of Oncology-Hematology, University of Nebraska Medical Center, Omaha, NE 68198-7680, USA; ^2^Department of Internal Medicine, Creighton University Medical School, Omaha, NE 68131, USA

## Abstract

*Background*: This study investigated the frequency of patients with HCC who refused cancer-directed surgery and the characteristics and outcomes of these patients. *Patients and Methods*: A retrospective study was performed using data from the Surveillance, Epidemiology, and End Results (SEER) Program. Characteristics of patients who refused CDS were compared with those who accepted surgery using logistic regression. The effect of refusing CDS on mortality was evaluated by Cox proportional hazards analysis. *Results*: Among 4373 surgical candidates, 142 patients (3.2%) refused the recommended CDS. The patients who refused CDS were frequently older, African American, widowed or divorced, and had advanced-stage tumors. In a logistic regression analysis, older age, African American, and being divorced or widowed were independently associated with refusal of CDS. After adjusting for other patient and tumor characteristics, the patients who refused CDS had a 2.5-fold (95% confidence interval, 2.339–3.189) higher risk of dying from HCC in comparison with patients who had CDS. Conclusions: The high rate of refusal may contribute in part to the disparity in utilization of CDS. Of greatest concern is that the patients who declined CDS had an impaired survival. This information might be helpful for patients to make a better-informed decision.

## 1. Introduction

Hepatocellular carcinoma (HCC) is the most frequent primary liver cancer. The annual number of new cases worldwide is approximately 550,000, representing more than 5% of human cancers and the third leading cause of cancer-related deaths [1, 2]. 

The goal of cancer-directed surgery (CDS) is to completely remove localized tumors or reduce the size of large tumors. CDS includes the curative surgeries (such as hepatectomy and transplantation) and local regional therapy, which employ modern surgical tools (including laser, high-frequency electrical currents, radiation, and liquid nitrogen), sometimes in conjunction with alcohol or chemotherapy agents. Resection and transplantation offer the best chance of long-term, disease-free survival and overall survival for patients with HCC. In a large series of patients from specialty institutions, the median survival of those who underwent resection of HCC lesions has ranged from 30 to 70 months [2–6]. Local regional therapy can provide alternatives for patients who are not candidate for resection or transplantation. Recently, chemoembolation has demonstrated survival benefits [7–11]. The 5-year survival rates for patients who underwent surgery, transhepatic arterial embolization (TAE), or supportive treatment were 43.6%, 25.6%, and 3.7%, respectively [5]. 

Significant advances in preoperative evaluation, surgical techniques, and postoperative care have reduced perioperative morbidity and mortality associated with liver surgery [11–18]. However, not all patients agree to the CDS recommended by their surgeon, even when it is potentially lifesaving or life prolonging. Patients who face the option of surgical interventions must examine/weigh the potential tradeoffs between benefits and burdens. For some patients surgery remains an invasive, risky intervention; thus, they may choose not to be operated on. Although there has been a great deal of investigation into decision making by patients with cancer, remarkably, there is virtually no research on the frequency of HCC patients refusing CDS and the factors that influence their decisions. Understanding the factors underlying refusal can direct/guide strategies to improve patient satisfaction, surgical utilization, and outcome of cancer care. 

The aim of this population-based study was to assess the frequency of refusal of CDS, identify the characteristics of patients who decide to refuse the recommended CDS for HCC, and estimate the impact of this decision on disease-specific survival.

## 2. Patients and Methods

### 2.1. Data Source and Cohort

The study cohort was composed of patients registered to the National Cancer Institute Surveillance, Epidemiology, and End Results (SEER) Program from 1985 to 2004. The SEER program collects uniformly reported data from 17 population-based cancer registries covering approximately 26.2% of the US population [19]. 

All adult patients with a microscopically confirmed diagnosis of HCC between 1985 and 2004 were identified using International Classification of Diseases for Oncology, third edition, histology codes 8170 (HCC) in combination with site code C22.0 (liver). We excluded the cases that were diagnosed at autopsy or on the basis of death certificates only as well as patients with multiple primaries. 

Demographic information recorded for each patient included age, sex, race/ethnicity, marital status, and year of diagnosis. These data were also included in multivariate analyses as covariates. Cancer-specific data evaluated for each patient included stage at presentation and histology grade. The tumor stage was evaluated using the SEER historic staging system (localized, regional, and distant). 

Cancer-directed surgery (CDS) was defined in the SEER database as any treatment that is given to modify, control, remove, or destroy primary or metastatic cancer tissue. These treatments included surgical resection (hepatectomy), transplantation, and local regional therapy (such as radiation frequency ablation, chemoembolization, and embolization) based on values for site-specific surgery and codes for the surgery of the primary site within the database [19]. 

The SEER database collected data from the clinical files of all HCC patients who were recommended or not recommended to undergo CDS; we differentiated between those who refused surgery and those who did not have surgery for other reasons. Whether patients underwent CDS (i.e., surgery intended for cure) was noted, the reason for not undergoing CDS was classified as “not recommended”, “contraindicated”, “refused”, or “unknown”. SEER collects this variable directly from the patient's medical record. “Not recommended” is coded if the physician believed that surgery was not the best treatment option, “contraindicated” is coded if a medical contraindication exists, and “refused” is coded if the patient refused surgery [19].

### 2.2. Statistical Analysis

Cancer-specific survival was studied by the actuarial method. Kaplan and Meier estimates of survival [20] and Cox proportional hazards model [21] were used to evaluate the impact that potential prognostic variables have on survival. Statistical analyses were performed with SPSS software (SPSS 10 version, Chicago, IL). 

 This study was approved by the University of Nebraska Medical Center Institutional Review Boards.

## 3. Results

### 3.1. Patient and Tumor Characteristics and Frequency of Cancer-Directed Surgery Refusal

A total of 8806 adult patients with localized HCC were identified in the SEER Registry between January 1985 and December 2004; the mean age of the cohort was 64 ± 13 years old (median age is 64 years, with a range of 18 to 104 years). The majority of patients were white, accounting for 5532 patients (63.3%). There were 947 African American patients (10.8%), and the other ethnic groups comprised 2266 patients (25.9%). 

 Of the entire cohort, 4373 (49.7%) were recommended to undergo CDS, while 4372 (49.6%) patients were determined not to be surgical candidates. No information was provided for the rest of the 61 (0.7%) patients.  Table 1 provides detailed information about the demographics, tumor characteristics, and treatments of the 4373 CDS candidates and 4372 noncandidates. The surgical candidates were on average three years older (62 ± 13 years) than the nonCDS candidates (65 ± 13 years) and more likely to be married. 

Among the 4373 surgical candidates, 3016 (68.9%) patients eventually had CDS performed; 142 (3.2%) patients declined the recommended CDS; 1125 (25.7%) patients did not undergo CDS for unknown reasons; 79 (1.8%) patients had no information regarding whether the recommended CDS was performed; and the remaining 11 (0.2%) patients died prior to their planned CDS.

### 3.2. Factors Associated with Surgical Candidate Recommendations/Selection

In a logistic regression analysis, age, marital status, tumor grade, size, and later year of diagnosis were found significantly correlated with being recommended to have CDS. Younger age and being married were independent predictors of selection for CDS. There was no racial difference between CDS candidates and noncandidates (Table 3).

### 3.3. Factors Associated with Refusal of Cancer-Directed Surgery

Table 2 presents the demographic and clinical characteristics of the patients who refused CDS (*N* = 142) or accepted and underwent CDS (*N* = 3016) and those patients did not undergo CDS for unknown reasons (*N* = 1125). The patients who refused surgery were on average eight years older (68 ± 13 years) than the patients who accepted CDS (60 ± 12 years). They were also more frequently nonCaucasian. The tendency to refuse CDS was significantly higher in those divorced and widowed. The demographic and clinical characteristics of the patients who, refused CDS and those patients did not undergo CDS for unknown reasons are very similar. 

Table 4 presents the results of a multivariable analysis of factors associated with refusal of CDS. The results of the analysis showed that older age, African American, being divorced, or being widowed and larger tumor size were independently associated with refusal of CDS.

### 3.4. Refusal of Cancer-Directed Surgery and Mortality

Table 5 presents the results of multivariate survival analyses using the Cox proportional hazards model. Refusal of CDS was identified as an independent factor for cancer-specific mortality. The risk of dying of HCC was increased by 2.5 fold (hazard ratio, 2.5; 95% confidence interval, 2.046–3.013) among patients who refused CDS in comparison with those who accepted CDS. Older age, male gender, African American, being widowed, and having a higher-grade tumor and an earlier diagnosis year were also independently associated with a higher cancer-specific mortality. 

**Figure 1:**
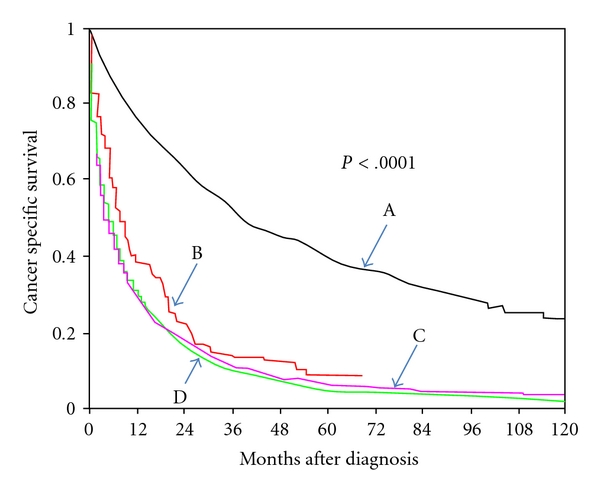
Cancer-specific survival in patients with HCC who refused or underwent cancer-directed surgery. Cancer-specific survival curve: A, CDS; B, CDS refused; C, CDS recommended but not performed; D, noncandidates for CDS.

Figure 1 shows the cancer-specific survival curves of patients refusing and patients accepting CDS. The patients with localized HCC who refused CDS had a survival similar to those who were considered nonsurgical candidates.

## 4. Discussion

The use of CDS as a cornerstone treatment of early-stage HCC has evolved over the last 20 years; significant advances in preoperative evaluation, surgical techniques, and postoperative care have reduced the perioperative morbidity and mortality associated with liver surgery [11–15]. Mortality after hepatectomy has dropped from approximately 25% in the 1960s to less than 3% today and investigators from high-volume centers report 0% mortality [18]. Hepatectomy and liver transplantation remain the only potentially curative therapy for localized liver cancer [5, 6]. Furthermore, several multi-institutional randomized clinical trials have demonstrated the safety and efficacy of local regional surgery (radiofrequency and chemoembolization) in the management of HCC [6–10]. 

This study is the first to quantify how often CDS is refused and the first to examine the common features of patients with HCC who refuse CDS and the impact that their refusal has on cancer-specific survival. We found 3.2% of the surgical candidates refused a cancer-directed surgical intervention. This result is compatible with previous findings [20, 22]. Our results clearly demonstrate that the risk of dying from HCC is more than doubled for patients who refuse CDS compared to those who undergo the recommended CDS, regardless of demographic factors, tumor grade, and stage. 

In our study, the older, unmarried patients were less likely to be recommended CDS and more likely to refuse CDS if it was offered than their younger, married counterparts. Although race was not associated with selection for CDS, African American patients were more likely to refuse a surgical intervention than their Caucasian counterparts. Physicians should be aware that these patients are at an increased risk to refuse surgery. For this population in particular, surgeons should strive for effective communication with the patient and emphasize the important role of surgery in managing HCC. 

Elderly patients tend to receive less optimal therapy than younger patients [23]; the reasons for this observation remain elusive. Whether the reason is the physician's perceived high operative risk, the lack of long-term benefits in elderly patients, or the physician's greater investment in convincing younger patients of the benefit of CDS, the topic needs further investigation [23, 24]. Our finding of a higher rate of refusal in the elderly likely serves as another explanation for the underuse of CDS in this population. 

The impact of age on operative risk is controversial. In a study of elderly patients with HCC, the survival difference by age disappeared when patients were compared within each treatment group, suggesting a close link between undertreatment and shorter survival [25]; for the patients with good liver function and good performance status, aggressive treatment of HCC improved the survival rate, even in the extremely elderly patients [26, 27]. Hepatic resection and transcatheter arterial chemoembolization for HCC in elderly patients (>70 years) were well tolerated and led to an improved survival rate [7]. Age alone should not be considered a contraindication to liver surgery [2, 5, 6]. Because performance status and physiological age are more important than chronological age, elderly patients with HCC should be fully evaluated to select all patients who would potentially benefit from aggressive surgical strategies. 

Previous studies have shown that African Americans and Asians are also significantly less likely to receive a transplant [28]. Blacks were found 24–27% less likely to receive surgical therapy than white individuals. The racial disparities in utilizing surgical treatment and in survival were most striking between black and white patients with localized HCC [29]. In addition to their potentially reduced access to medical care, the high rate of refusal in black patients may explain, at least partially, the underuse of CDS in this population. The higher refusal rate may be attributable to cultural reasons, personal beliefs, different perceptions of surgery [30], and distrust of health care systems [8]. We found no significant difference in the selection of CDS candidates between African American and white patients; however, once recommended to have surgery, African Americans are more likely to refuse CDS. 

Few studies address the natural history of HCC, that is, the outcome of HCC without therapy [31, 32]. In our study, we found that refusal of CDS has a significant impact on the cancer-specific survival of patients with HCC. The survival of these patients was similar to the nonsurgical candidates. Since there was no effective chemotherapy for HCC during the study period, because CDS offers the best chance of survival for most HCC patients [1, 2], it should be evaluated carefully and offered to patients who may potentially benefit from it. 

Treatment refusal may be a marker for insufficient patient-centered decision making, as patients may refuse treatments that they perceive as inadequate in meeting their treatment goals. Our study suggests that surgeons may be recommending treatments that pose unacceptable burdens to some patients or that fail to meet the patients' goals; we found old age is one of the greatest risk factors of refusing CDS. Given the rapidly aging population, further studies should focus on identifying specific reasons why these patients refuse CDS. The new breakthrough of therapy with signal transduction inhibitors, such as sorafinib, can provide an alternative for those patients who refuse CDS [31]; however, patients need to be aware that this new therapy is clearly palliative. 

Our study has some limitations. First, decision making regarding surgery is a complex process and involves both care-provider and patient factors. Although we were able to create a cohort with a full range of demographic and clinical variables, we were unable to assess the patient's performance status, comorbidities, and other factors such as characteristics of the surgeon that might influence receipt or refusal of CDS. This study was also limited by the retrospective and nonrandomized nature of any registry-based study [33]; even after adjusting for all available variables linked to patient refusal or prognosis, we cannot rule out a selection bias related to unrecorded factors. Finally, the clinical information available from a registry is not as detailed as that from chart review. However, the use of specific survival rather than overall survival in our study has modified the limitation to some degree. 

Despite its limitations, our study adds to the knowledge of disparities cancer treatment related to age, race and marital status. First, the sample size was large enough for us to assess the patterns of care in a diverse population and to examine a number of potentially confounding variables. Second, our study may partially explain why the use of CDS varies by age and race. Further research should focus on the physician-patient encounter as a potential source of these disparities [31], allowing a better understanding of the underlying reason for refusal. Enhancing communication may be one of the simplest approaches to reduce the disparities in surgical treatment in cancer patients. Both patients and surgeons need to be educated in optimal HCC management. 

In summary, using a national population-based data sample, we found 3.2% of surgical candidates refused CDS for HCC. We compared the characteristics of these patients and investigated the impact of refusal on mortality. The higher rate of refusal may contribute in part to the underutilization of CDS in certain populations. To make a well-informed decision, patients should be informed of both the beneficial and adverse effects of the treatment options. The patients have full rights in their decision of cancer treatment. However, some patients who refuse CDS may miss out on the only prospect of prolonging their lives or even on the opportunity of being cured. The findings of this study may be helpful for those patients who are facing this therapeutic decision.

## Figures and Tables

**Table 1:** Demographic and clinical characteristics of patients with localized HCC. CDS: cancer-directed surgery.

Group	CDS candidates	NonCDS candidates	*P*
	*N* = 4373	*N* = 4372	
Age (mean ± SD)	65 ± 13	62 ± 13	<.001
Age group			<.001
<60	1924 (44.0%)	1466 (33.5%)	
≥60	2449 (56.0%)	2906 (66.5%)	
Gender			.59
Male	3137 (71.7%)	3174 (72.6%)	
Female	1236 (28.3%)	1198 (27.4%)	
Race			.74
White	2786 (63.7%)	2746 (62.8%)	
Black	454 (10.4%)	493 (11.3%)	
Asian	1050 (24.0%)	1072 (24.5%)	
American Indian	62 (1.4%)	43 (1.0%)	
Unknown	21 (0.5%)	18 (0.4%)	
Marital status			.001
Married	2715 (62.1%)	2400 (54.9%)	
Divorced	406 (9.3%)	456 (10.4%)	
Separated	44 (1.0%)	54 (1.2%)	
Single	581(9.8%)	678 (15.5%)	
Windowed	473 (10.8%)	653 (14.9%)	
Unknown	154 (3.5%)	131 (3.0%)	
Grade			<.001
Well differentiated	1043 (23.9%)	617 (14.1%)	
Moderately differentiated	1025 (23.4%)	428 (9.8%)	
Poorly differentiated	378 (8.6%)	323 (7.4%)	
Undifferentiated	50 (1.1%)	37 (0.8%)	
Unknown	1877 (42.9%)	2967 (39.1%)	
Tumor size			.01
<5 cm	206 (4.7%)	236 (5.4%)	
≥5 cm	383 (8.8%)	315 (7.2%)	
Unknown	3784 (86.5%)	3821 (87.4%)	
Year of diagnosis			.46
1985–1994	663 (15.2%)	638 (14.6%)	
1995–2004	3710 (84.8%)	3734 (85.4%)	

**Table 2:** Comparison of patients with localized HCC who had, with those who, refused CDS, and those CDS were not performed due to unknown reason. CDS: cancer-directed surgery.

	Refused	CDS	Not performed
	*N*(%)	*N*(%)	*N*(%)
Age (mean ± SD)	68 ± 13	60 ± 12	66 ± 13
Age group			
<60	35 (24.6)	1494 (49.5)	354 (31.5)
≥60	107 (75.5)	1522 (50.5)	771 (68.5)
Gender			
Male	99 (69.7)	2139 (70.9)	827 (73.5)
Female	43 (30.3)	877 (29.1)	298 (26.5)
Race			
White	79 (55.6)	1875 (62.2)	778 (69.2)
Black	19 (13.4)	267 (8.9)	157 (14.0)
Asian	40 (28.2)	826 (27.4)	159 (14.1)
American Indian	4 (2.8)	33 (1.1)	25 (2.2)
Unknown	0 (0.0)	15 (0.5)	6 (0.5)
Marital status			
Married	72 (50.7)	1976 (65.5)	610 (54.2)
Divorced	18 (12.8)	258 (8.6)	121 (10.8)
Separated	0 (0.0)	37 (1.2)	7 (0.6)
Single	15 (10.6)	395 (13.1)	156 (13.9)
Windowed	28 (19.7)	272 (9.0)	168 (14.9)
Unknown	9 (6.3)	78 (2.6)	63 (5.6)
Grade			
Well differentiated	19 (13.4)	801 (26.6)	205 (18.2)
Moderately differentiated	17 (12.0)	875 (29.0)	125 (11.1)
Poorly differentiated	9 (6.3)	282 (9.4)	78 (6.9)
Undifferentiated	0 (0.0)	39 (1.3)	10 (0.9)
Unknown	97 (68.3)	1019 (33.8)	707 (62.9)
Tumor size			
<5 cm	5 (3.5)	364 (12.1)	11 (1.0)
≥5 cm	8 (5.6)	171 (5.7)	23 (2.0)
Unknown	129 (90.8)	2481 (82.3)	1091 (97.0)
Year of diagnosis			
1985–1994	23 (16.2%)	345 (11.4)	290 (25.8)
1995–2004	119 (83.8)	2671 (88.6)	835 (74.2)

**Table 3:** Logistic regression analysis of factors associated with recommendation of cancer-directed surgery in patients with localized HCC.

Characteristics	Group	OR	95% Cl	*P*-value
Age	<60	1.00		
	≥60	0.606	0.551–0.667	<.001

Gender	Female	1.00		
	Male	0.816	0.735–0.906	<.001

Ethnicity	White	1.00		
	Black	0.920	0.794–1.066	.268
	Others	0.962	0.868–1.067	.465

Marital status	Married	1.00		
	Widowed	0.696	0.602–0.805	<.001
	Divorced/separated	0.731	0.631–0.847	<.001
	Singled	0.705	0.616–0.806	<.001
	Other	1.016	0.791–1.304	.903

Grade	Low grade	1.00		
	High grade	0.609	0.518–0.716	<.001
	Unknown	0.319	0.290–0.351	<.001

Tumor size	<5 cm	1.00		
	≥5 cm	0.398	0.310–0.510	<.001
	Unknown	0.652	0.546–0.779	<.001

Year of diagnosis	1985–1994	1.00		
	1995–2004	0.841	0.742–0.954	.007

OR = odds ratio; CI = confidence interval.

**Table 4:** Logistic regression analysis of factors associated with refusal of cancer-directed surgery in patients with localized HCC.

Characteristics	Group	OR	95% Cl	*P*-value
Age	<60	1.00		
	≥60	2.901	1.921–4.383	<.001

Gender	Female	1.00		
	Male	1.445	0.961–2.173	.077

Ethnicity	White	1.00		
	Black	1.836	1.065–3.168	.029
	Others	1.172	0.791–1.735	.429

Marital status	Married	1.00		
	Widowed	2.395	1.438–3.990	.001
	Divorced/separated	1.947	1.110–3.417	.020
	Singled	1.199	0.664–2.165	.548
	Other	3.128	1.466–6.677	.003

Grade	Low grade	1.00		
	High grade	1.552	0.733–3.284	.250
	Unknown	4.432	2.983–6.586	<.001

Tumor size	<5 cm	1.00		.017
	≥5 cm	4.036	1.277–12.755	
	Unknown	3.595	1.443–8.957	

Year of diagnosis	1985–1994	1.00		
	1995–2004	0.859	0.530–1.393	.538

OR = odds ratio; CI = confidence interval.

**Table 5:** Cox proportional hazards model of factors associated with cancer-specific mortality in patients with localized HCC.

Characteristics	Group	HR	95% Cl	*P*-value
Age	<60	1.00		
	≥60	1.223	1.156–1.295	<.001

Gender	Female	1.00		
	Male	1.132	1.067–1.202	<.001

Ethnicity	White	1.00		
	Black	1.145	1.055–1.242	.001
	Others	0.828	0.780–0.880	<.001

Marital status	Married	1.00		
	Widowed	1.220	1.128–1.319	<.001
	Divorced/separated	1.027	0.943–1.118	.540
	Singled	1.054	0.976–1.139	.181
	Others	1.131	0.983–1.300	.084

Grade	Low grade	1.00		
	High grade	1.608	1.465–1.766	<.001
	Unknown	1.262	1.190–1.338	<.001

Tumor size	<5 cm	1.00		
	≥5 cm	1.978	1.556–2.515	<.001
	Unknown	1.942	1.593–2.366	<.001

Diagnosis year	1989–2004	1.00		
	1973–1988	0.741	0.695–0.791	<.001

CDS	Performed	1.00		
	Refused	2.444	2.014–2.966	<.001
	Nonsurgical candidate	3.215	3.003–3.441	<.001

HR = hazard ratio; CI = confidence interval; CDS: cancer-directed surgery.
